# Editorial: Advanced quantitative indexes in cardiovascular magnetic resonance imaging

**DOI:** 10.3389/fcvm.2024.1302397

**Published:** 2024-02-02

**Authors:** Xiaoyue Zhou, Yucheng Chen, Rob J. van der Geest, Peng Hu, Ming-Yen Ng

**Affiliations:** ^1^ MR Collaboration, Siemens Healthineers Ltd., Shanghai, China; ^2^ Department of Cardiology, West China Hospital, Sichuan University, Chengdu, China; ^3^ Department of Radiology, Leiden University Medical Center (LUMC), Leiden, Netherlands; ^4^ School of Biomedical Engineering, ShanghaiTech University, Shanghai, China; ^5^ Department of Diagnostic Radiology, School of Clinical Medicine, Li Ka Shing Faculty of Medicine, The University of Hong Kong, Pokfulam, Hong Kong SAR, China

**Keywords:** CMR, T1 mapping, 4D flow cardiac MR, strain analysis, quantitative image analysis

**Editorial on the Research Topic** Advanced quantitative indexes in cardiovascular magnetic resonance imaging

Cardiovascular Magnetic Resonance Imaging (CMRI) is widely utilized for diagnosing various heart diseases in routine clinical practice, providing information on heart morphology, function, and myocardial tissue characterization. Furthermore, CMRI demonstrates its capabilities in disease prognosis and guiding treatment planning, owing to its non-invasive and radiation-free features, making it applicable for follow-up studies. With the development of both MRI sequence techniques and image post-processing algorithms, CMRI has shifted its focus from conventional qualitative methods to novel quantitative approaches, offering fruitful advanced quantitative indexes, such as myocardial strain, T1/T2/T2* mapping, hemodynamics, and more. However, the reliability, accuracy, and, most importantly, the diagnostic and prognostic value of these advanced quantitative indexes obtained from CMRI still needs further improvement and exploration. Within the papers collected in this Research Collection, we see examples of technical/diagnostic/prognostic improvements of these quantitative methods, demonstrations of potential clinical applications and, reviews that highlight the current state-of-the-art in this field.

Myocardial strain has proven to be a more sensitive index than Left Ventricular Ejection Fraction (LVEF) in capturing early changes in heart function (1). Among various strain analysis methods, Displacement Encoding with Stimulated Echoes (DENSE) (2) and Feature Tracking (FT) (1, 3) emerge as the two most promising approaches. Efforts have been dedicated to reducing the scan time of the DENSE sequence and simplifying its post-processing, making it practical for integration into clinical routine protocols (4). Meanwhile, the accuracy and reliability of the method is also important and should be further studied. In this collection, Ghadimi et al. propose a regularized spatiotemporal least squares method for DENSE image post-processing. It provides more realistic measurements and improves the accuracy of the strain calculation from DENSE images. Such improvements can bolster diagnostic confidence and enhance the diagnostic efficiency for various heart diseases with myocardial dysfunction. Similarly, the reliability of strain measurements by FT largely depends on the sequence method used for MR cine acquisition. The recent adoption of Compressed Sensing (CS) has significantly accelerated the acquisition time of MR cine sequences, resulting in reduced breath-hold times and increased spatial/temporal resolution. Chen et al. investigate the impact of the strain value derived from both the conventional and CS based MR cine images. Their study reveals that FT based global strain analysis shows comparable results between the two sequences, while segmental strain exhibits remarkable variation between the two sequences. This suggests there is still room for improvement in both sequence and post-processing algorithms. Earl et al. explore the role of various strain analysis methods in the diagnosis of Duchenne muscular dystrophy, concluding that among these methods, Deep Learning (DL) holds the most promise for rapid and automated cardiac strain analysis. With the application of CS and DL, we anticipate the future will be characterized by fast, free-breathing imaging, and DL-based automated strain assessment.

Myocardial tissue characterization using Late Gadolinium Enhancement (LGE) is another advantage of CMRI. LGE is known as the gold-standard imaging method for diagnosing myocardial fibrosis. However, it is a qualitative imaging method in assessing myocardial tissue injury and is always limited to global descriptors. Innovatively, Jiang et al. propose an LGE pattern measurement based on the ratio of LGE to Left Ventricle (LV) mass, turning the LGE measurement into quantitative method and demonstrating its ability in prognosing cardiac function of fulminant myocarditis by categorizing the LGE images into two distinct patterns. More interestingly, Duchateau et al. explore a pixel-wise statistical analysis to reveal different regional lesion patterns in the LGE images, empowering quantitative diagnosis of subtle differences from the LGE images.

T1 mapping, compared to LGE, is known as a more sensitive index in the diagnosis of early myocardial changes and is widely used in clinical routine. Liu et al. show that both strain and T1 mapping offer advantages in detecting early-stage heart dysfunction in Danon disease. In another study, Jia et al. investigate myocardial involvement in chronic kidney patients, revealing that myocardial mapping exhibits higher diagnostic sensitivity than conventional LV indexes in differentiating chronic kidney disease stage. The advantage of T1 mapping is apparent especially in detecting diffuse fibrosis. However, cardiac and respiratory motion limits its application. Motion free T1 mapping techniques are still under developing. Sheagren et al. present a comprehensive review of the state-of-the-art motion-compensated T1 mapping methods. They point out that the potential future directions are in motion-resolved free-breathing parametric mapping and three-dimensional mapping techniques. Moreover, stress CMRI has garnered increased attention for its ability to identify early heart function deterioration, quantify myocardial blood flow and diagnose coronary microvascular dysfunction. Ma et al. explore the stress T1 response in type 2 diabetes mellitus rabbits, revealing a high correlation between stress T1 and the histopathologic measures. This finding holds promise for the detection of micro myocardial changes.

Fat deposition often occurs in many heart diseases, including type 2 diabetes mellitus, making fat quantification valuable for assessing disease progression. In the study by Edin et al., the association between regional fat deposition and cardiac remodeling is explored, revealing a connection between epicardial fat and impaired LV diastolic function. However, conventional fat quantification methods, such as MR spectroscopy (5) and quantitative Dixon (6–8), face challenges when applied to the myocardium due to cardiac and respiratory motion. Reliable fat quantification method is needed to obtain myocardial fat fraction in the future.

Four-dimensional flow offers abundant advanced quantitative indexes which aid in the non-invasive assessment of *in vivo* blood flow changes. Among them, Kinetic Energy (KE) is considered as an advanced quantitative index in 4D flow analysis reflecting the intensity of blood flow velocity fluctuations. It is often used for the quantitative assessment of blood flow dynamics in cardiac intracavitary areas or vessels. Besides, some cardiac pathologies may remain asymptomatic under rest conditions but can be revealed under stress conditions. In the study by Riva et al., changes in intra-cardiac blood flow KE between rest and stress are examined in a healthy cohort. The findings suggest that LV intracavitary flow dynamics can adapt to stress conditions. It may be further extended to assess hemodynamic changes in various heart diseases.

The utility of advanced quantitative CMRI indexes plays a crucial role in differential diagnosis and raising diagnostic confidence. Many heart diseases presenting with similar imaging appearances can be differentiated by the combination of advanced quantitative indexes. Yue et al. combine strain analysis with extracellular volume to differentiate early-stage amyloidosis from hypertrophic cardiomyopathy, which the two diseases often show very similar features in conventional CMRI. It is recognized that the Left Atrium (LA) function change may remodulate LV diastolic filling. Therefore, there may be strong correlations between quantitative indexes of LA and LV, termed ‘LA-LV couplings’. Conventional CMRI is always difficult to distinguish hypertrophic cardiomyopathy and hypertension in their early stage as their morphologic features are very similar. However, the LA and LV functional change of the two diseases may be different, which may be reflected by LA-LV couplings. Li et al. find that the LA-LV couplings are quite different in the two diseases and may enhance our understanding of the disease mechanisms. Another study by McGettrick et al. combine the eccentricity index derived from cardiac geometry and pulmonary artery distensibility to differentiate subtypes of chronic thromboembolic diseases. It shows that CMR parameters can be used in conjunction with other invasive and non-invasive investigations to evaluate patients who are suspected as having pulmonary vascular disease. Multi-parametric analysis in CMRI provides deeper insight into disease mechanisms. Lu et al. investigate the dominant factor of LV systolic dysfunction in amyloid using T1 mapping, first-pass perfusion, and LVEF, revealing amyloid overload as a major contributor of LV systolic dysfunction. Radiomics is another direction that can be integrated with CMRI, generating many novel quantitative indexes. He et al. utilize radiomic analysis to further excavate ventricular remodeling information from cine images, finding outstanding predictive effectiveness of adverse remodeling for patients with severe aortic stenosis after TAVR. A “2 out of 2” approach is used in the updated Lake Louise Criteria for myocardial inflammation diagnosis (9), highlighting the clinical efficiency of T1 and T2 mapping indexes. Pedersen et al. apply it in the study of myocardial inflammation after vaccination for SARS-CoV-2, showcasing its diagnostic capabilities. Filomena et al. provide an overview of the role of MRI in assessing cardiovascular inflammation. They conclude that CMR can help in revealing pathophysiological characteristics of inflammatory cardiomyopathy and may provide deeper insight into the disease with future technique developments. The strength of combining multiple advanced quantitative CMRI indexes is evident, positioning CMRI as a comprehensive and non-invasive one-stop-shop imaging modality in clinical routine.

In summary, we have seen many promising technique developments and apparent clinical evidence with the use of various advanced quantitative indexes obtained from CMRI. We are also aware of the gaps between these newly emerged quantitative methods and the diagnostic confidence and efficiency of using them in the clinical routine. Further standardization and validation of these indexes across diverse populations, sites, vendors are quite urgent and necessary. Looking ahead, we believe that fast, accurate, and simple quantitative methods will predominate the future of CMRI.

## References

[1] Xu J Yang W Zhao S Lu M. State-of-the-art myocardial strain by CMR feature tracking: clinical applications and future perspectives. Eur Radiol. (2022) 32(8):5424–35. 10.1007/s00330-022-08629-2 35201410

[2] Kim D Gilson WD Kramer CM Epstein FH. Myocardial tissue tracking with two-dimensional cine displacement-encoded MR imaging: development and initial evaluation. Radiology. (2004) 230:862–71. 10.1148/radiol.2303021213 14739307

[3] Pedrizzetti G Claus P Kilner PJ Nagel E. Principles of cardiovascular magnetic resonance feature tracking and echocardiographic speckle tracking for informed clinical use. J Cardiovasc Magn Reson. (2016) 18:51. 10.1186/s12968-016-0269-7 27561421 PMC5000424

[4] Chen X Yang Y Cai X Auger DA Meyer CH Salerno M Accelerated two-dimensional cine DENSE cardiovascular magnetic resonance using compressed sensing and parallel imaging. J Cardiovasc Magn Reson. (2016) 18:38. 10.1186/s12968-016-0253-2 27301487 PMC4906684

[5] Venkatesh BA Lima JAC Bluemke DA Shenghan L Steenbergen C Chia-Ying L. MR proton spectroscopy for myocardial lipid deposition quantification: a quantitative comparison between 1.5-T and 3-T. J Magn Reson Imaging. (2012) 36(5):1222–30. 10.1002/jmri.23761 22826193 PMC3482140

[6] Farrelly C Shah S Davarpanah A Keeling AN Carr JC. ECG-gated multiecho dixon fat-water separation in ardiac MRI: advantages over conventional fat-saturated imaging. AJR. (2012) 199:W74–83. 10.2214/AJR.11.7759 22733934

[7] Lu M Zhao S Jiang S Yin G Wang C Zhang Y Fat deposition in dilated cardiomyopathy assessed by CMR. JACC Cardiovasc Imaging. (2013) 6(8):889–98. 10.1016/j.jcmg.2013.04.010 23850250

[8] Ng ACT Strudwick M van der Geest RJ Ng ACC Gillinder L Goo SY Impact of epicardial adipose tissue, left ventricular myocardial fat content, and interstitial fibrosis on myocardial Contractile function. Circ Cardiovasc Imaging. (2018) 11(8):e007372. 10.1161/CIRCIMAGING.117.007372 30354491

[9] Ferreira VM Schulz-Menger J Holmvang G Kramer CM Carbone I Sechtem U Cardiovascular magnetic resonance in nonischemic myocardial inﬂammation: expert recommendations. J Am Coll Cardiol. (2018) 72:3158–76. 10.1016/j.jacc.2018.09.072 30545455

